# Antimetastatic Effects of Norcantharidin on Hepatocellular Carcinoma by Transcriptional Inhibition of MMP-9 through Modulation of NF-kB Activity

**DOI:** 10.1371/journal.pone.0031055

**Published:** 2012-02-07

**Authors:** Chao-Bin Yeh, Ming-Ju Hsieh, Yi-Hsien Hsieh, Ming-Hsien Chien, Hui-Ling Chiou, Shun-Fa Yang

**Affiliations:** 1 Department of Emergency Medicine, School of Medicine, Chung Shan Medical University, Taichung, Taiwan; 2 Department of Emergency Medicine, Chung Shan Medical University Hospital, Taichung, Taiwan; 3 School of Medical Laboratory and Biotechnology, Chung Shan Medical University, Taichung, Taiwan; 4 Department of Biochemistry, School of Medicine, Chung Shan Medical University, Taichung, Taiwan; 5 Wan Fang Hospital, Taipei Medical University, Taipei, Taiwan; 6 Graduate Institute of Clinical Medicine, Taipei Medical University, Taipei, Taiwan; 7 Institute of Medicine, Chung Shan Medical University, Taichung, Taiwan; 8 Department of Medical Research, Chung Shan Medical University Hospital, Taichung, Taiwan; Ospedale Pediatrico Bambino Gesu', Italy

## Abstract

**Background:**

The rate of morbidity and mortality of hepatocellular carcinoma (HCC) in Taiwan has not lessened because of difficulty in treating tumor metastasis. Norcantharidin (NCTD) is currently used as an anticancer drug for hepatoma, breast cancer, and colorectal adenocarcinoma. NCTD possesses various biological anticancer activities, including apoptosis. However, detailed effects and molecular mechanisms of NCTD on metastasis are unclear. Thus, HCC cells were subjected to treatment with NCTD and then analyzed to determine the effects of NCTD on cell metastasis.

**Methodology/Principal Findings:**

Modified Boyden chamber assays revealed that NCTD treatment inhibited cell migration and invasion capacities of HCC cells substantially. Results of zymography and western blotting showed that activities and protein levels of matrix metalloproteinase-9 (MMP-9) and urokinase plasminogen activator (u-PA) were inhibited by NCTD. Western blot analysis showed that NCTD inhibits phosphorylation of ERK1/2. Testing of mRNA level, quantitative real-time PCR, and promoter assays evaluated the inhibitory effects of NCTD on MMP-9 and u-PA expression in HCC cells. The chromatin immunoprecipitation (ChIP) assay for analyzing the genomic DNA sequences bound to these proteins was reactive to the transcription protein nuclear factor (NF)-kappaB, which was inhibited by NCTD. The expression of NF-kappa B was measured by western blot analysis, which revealed decreased nuclear-factor DNA-binding activity after NCTD treatment.

**Conclusions:**

NCTD inhibited MMP-9 and u-PA expression through the phosphorylation of ERK1/2 and NF-kappaB signaling pathway which serves as a powerful chemopreventive agent in HCC cell metastasis.

## Introduction

Hepatocellular carcinoma (HCC) is a common malignant neoplasm and cause of cancer-related death in Asian countries. The mortality rate of HCC in Taiwan has not decreased, mainly because of the difficulty of treatment related to invasion and metastasis [1]. Generally, metastasis of cancer cells involves multiple processes and various cytophysiological changes, including altered adhesive capability between cells and the extracellular matrix (ECM) and damaged intercellular interaction. Degradation of ECM by cancer cells through proteases such as serine proteinase, matrix metalloproteinases (MMPs), cathepsins, and plasminogen activator (PA) may lead to the separation of the intercellular matrix to promote cancer cell mobility, and may eventually lead to metastasis. Among the involved proteases, MMP-2, MMP-9, and u-PA are most vital for the degradation of base membranes, and are therefore deeply involved in cancer invasion and metastasis [2]–[3]. Various factors such as growth factors, cytokines, certain chemicals, or even physical stimulation may promote MMP expression, whereas TGF-b, retinoic acids, and glucocorticoids may inhibit MMPs. In addition, u-PA or tissue-type plasminogen activator (t-PA) may activate a series of protein degradation reactions to regulate or activate MMPs. MMP activity is prone to the inhibition of endogenous tissue by metalloproteinases (TIMPs), which are specific inhibitors of MMPs, and the imbalance between MMPs and TIMPs may contribute to ECM degradation or deposition [3], [4].

Cantharidin and norcantharidin (NCTD, exo-7-oxabicylo-[2.2.1] heptane-2,3-dicarboxylic anhydride) are known to possess anticancer activities because they suppress the activity of serine/threonine protein phosphates [5], [6]. In our previous study, the structure-activity relationship (SAR) of cantharidin analogues suggested that anhydride ether oxygen in these analogues may correlate with HCC survival suppression, and the elimination of bridging ether oxygen on the ring can decrease cytotoxicity. However, cantharidin is unsuitable for cancer therapy because of its high cytotoxicity *in vitro* [IC (50) = 21 µM in primary cultured rat hepatocytes] [7]. The demethylated analogue of cantharidin is NCTD, which reduces the toxicity of cantharidin and is a potential anticancer drug for various cancer cells. A recent study showed that an NCTD-Nd3II derivative possesses anti-hepatoma activity, both *in vitro* and *in vivo*. It exerts its antiproliferative activity through apoptosis, G2/M cell-cycle arrest, and regulation of cyclin B1/cdc-2, p21, and Bcl-2/Bax [8]. Yang et al. reported that NCTD induces apoptosis of breast cancer cells through activities of mitogen-activated protein kinases and signal transducers and activators of transcription. Consequently, NCTD may disturb cell-cycle distribution of breast cancer cells through p53- and Chk-related pathways [9]. Moreover, Liao et al. reported that NCTD induces cell cycle arrest and inhibits progression of human leukemic Jurkat T cells through mitogen-activated protein kinase-mediated regulation of interleukin-2 production [10]. Furthermore, Chang et al. showed that NCTD induced cytotoxicity in HepG2 cells by apoptosis, which is mediated through ROS generation and mitochondrial pathways [11], and a number of authors suggested that NCTD demonstrates anti-proliferative effects on human HepG2 cells in cell cultures [12]. NCTD thus inhibits the cell growth of various cancers by inducing apoptosis in cancer cells [13]–[21]. Research has confirmed that NCTD not only induces apoptosis, but also shows an anti-metastasis effect in certain cancer cells. Luan et al. reported that NCTD could reduce human lung cancer cell growth and migration because of inhibitory effects in human lung cancer A549 cells [13]. Chen et al. showed that NCTD demonstrates an inhibitory effect on tumor invasion and metastasis in colorectal adenocarcinoma cells. NCTD possesses anti-angiogenic activity and could be useful in anticancer therapy as an anti-metastatic and anti-angiogenic agent in colorectal adenocarcinoma CT26 cells [15], [22], [23]. Studies on the inhibitory effects of NCTD on HCC cell invasion and migration behavior, however, are no literature report, and these effects warrant further examination. Thus, we studied the effects, mechanisms, and pathway of NCTD in HCC metastasis.

## Results

### Effect of NCTD on the Viability of Huh7 Cells and Normal Hepatocytes

**Figure 1:**
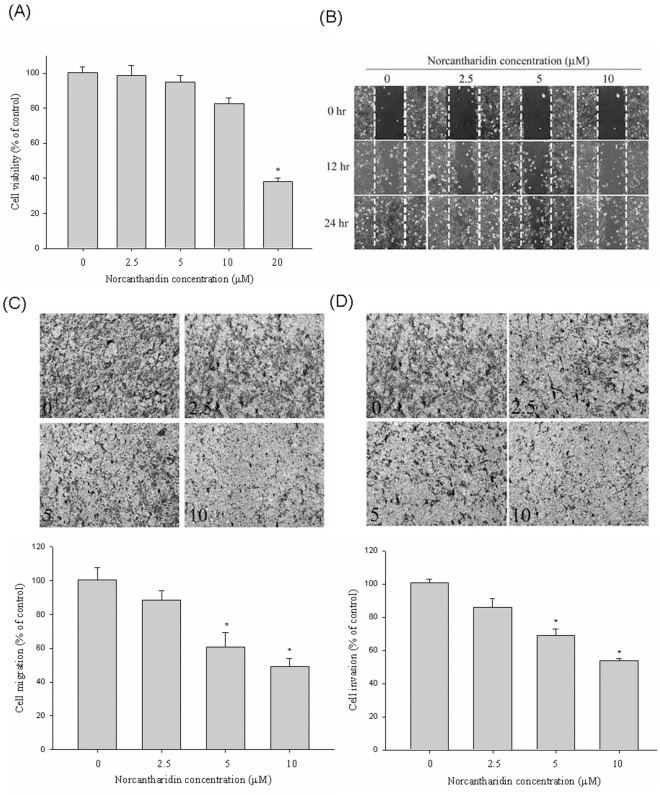
Effect of NCTD on cell viability, *in vitro* wound closure, cell migration and invasion in Huh7 cells. (A) Huh7 cells were treated with NCTD (0, 2.5, 5, 10 and 20 µM) for 24 h before being subjected to a MTT assay for cell viability. The values represented the means ± SD of at least three independent experiments. (B) Huh7 cells were wounded and then treated with vehicle (DMSO) or NCTD (2.5, 5 and 10 µM) for 0 h, 12 h and 24 h in 0.5% FBS-containing medium. At 0, 12 and 24 h, phase-contrast pictures of the wounds at three different locations were taken. (C & D) Figure C&D showing the cell migration and invasion were measured using a Boyden chamber for 16 h and 24 h with polycarbonate filters respectively. The migration and invasion abilities of Huh7 cells were quantified by counting the number of cells that invaded to the underside of the porous polycarbonate as described in the *Materials and Methods* section. The values represented the means ± SD of at least three independent experiments. *P<0.05 as compared with the vehicle group.

The cytotoxic effects of NCTD at various concentrations (0–20 µM) on Huh7 cells are shown in Fig. 1A. The MTT assay showed that, at the highest concentration of 20 µM, NCTD altered HCC cell viability, as previously described [7]. Thus, we used a concentration range of NCTD lower than this for all subsequent experiments. Furthermore, NCTD also did not alter the cell viability of normal hepatocytes (Figure S1).

We further investigated the effect of NCTD on the cell cycle regulation. Our results showed that treatment of Huh7 cells with NCTD (0–20 µM) for 24 h did not increase the incidence of apoptosis as evidenced by without significant changes in the sub G1 population between control and NCTD-treated Huh7 cells. Moreover, the proliferation rate of Huh7 cells was also not affected by NCTD due to the number of cells in S-phase was not changed significantly after treatment of NCTD for 24 h (Figure S2).

### Effects of NCTD on *In vitro* Wound Closure, Invasion, and Migration in Huh7 Cells

Figure 1B displays findings from a wound closure assay to determine the effects of NCTD on the migration of Huh7 cells. It shows representative photographs of Huh7 cells migrating into scratch wounds under treatment with NCTD. Figures 1C and 1D show the effect of NCTD on cell migration and invasion in Huh7 cells that were treated with 0, 2.5, 5, and 10 µM of NCTD for 16 h (cell migration) and 24 h (cell invasion). Using a cell migration and invasion assay with a Boyden chamber, we showed that NCTD reduced the invasion and migration of Huh7 cells substantially in a concentration-dependent manner. Similar anti-migration effect of NCTD was observed in SK-Hep1 HCC cell line (Figure S3).

### Effect of NCTD on the Protein Levels of MMPs and their Endogenous Inhibitors

**Figure 2:**
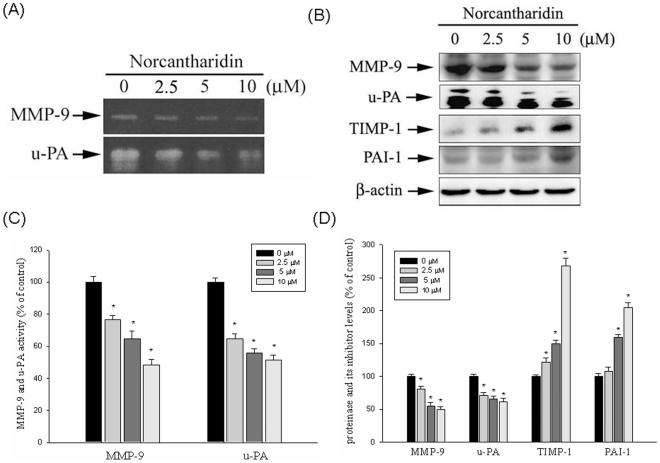
Effects of NCTD on the activity and protein level of MMP-9, u-PA and the protein level of the endogenous inhibitor TIMP-1 and PAI-1. (A) Huh7 cells were treated with NCTD (0, 2.5, 5 and 10 µM) for 24 h and then subjected to gelatin and casein zymography to analyze the activity of MMP-9 and u-PA respectively. (B) Huh7 cells were treated with NCTD (0, 2.5, 5 and 10 µM) for 24 h and then subjected to western blotting to analyze the protein levels of MMP-9, u-PA, PAI-1 and TIMP-1. (C&D) Quantitative results of MMP-9, u-PA, PAI-1 and TIMP-1 protein levels which were adjusted with β-actin protein level. The values represented the means ± SD of at least three independent experiments. *P<0.05 as compared with the vehicle group.

Huh7 cells were treated with NCTD (0, 2.5, 5, and 10 µM) for 24 h, and then subjected to gelatin zymography and casein zymography for analysis of MMP-9 and u-PA activity, respectively. As shown in Fig. 2A and 2C, NCTD treatment may lead to reduced activity of MMP-9 and u-PA in a dose-dependent manner. Similar effect on MMP-9 activity of NCTD was also observed in SK-Hep1 HCC cell line (Figure S4). Figure 2B shows a western blotting analysis of the protein levels of MMP-9, u-PA, PAI-1, and TIMP-1. MMP-9, u-PA, PAI-1, and TIMP-1 protein levels were adjusted with β-actin. Protein levels of MMP-9 and u-PA decreased significantly, whereas those of their respective inhibitors PAI-1 and TIMP-1 increased (Figure 2D). In addition to MMP-9, we found that NCTD also can inhibit the activation of focal adhesion kinase, FAK, significantly (Figure S5). This result suggested that the inhibition of FAK activation by NCTD might also contribute to the migration/invasion inhibition in the Huh7 cells.

### Effect of NCTD on the Transcriptional Level of MMP-9 and u-PA

**Figure 3:**
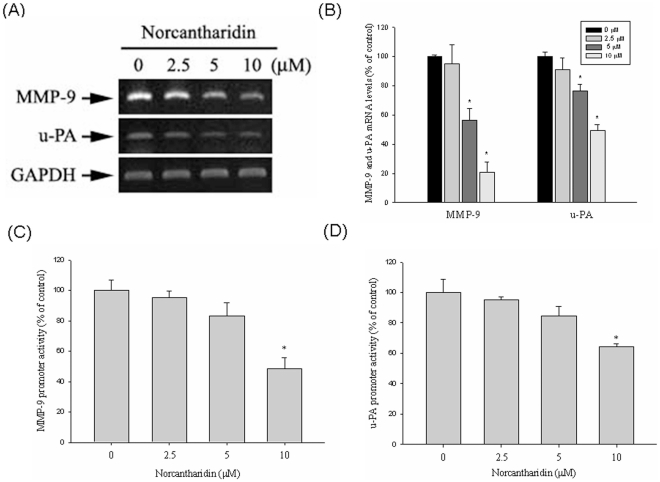
NCTD suppresses MMP-9 and u-PA expression at a transcriptional level. Huh7 cells were treated with NCTD (0, 2.5, 5 and 10 µM) for 24 h and then subjected to (A) Reverse Transcription-PCR and (B) quantitative real-time PCR to analyze the mRNA expression of MMP-9, or u-PA. (C&D) MMP-9 and u-PA promoter reporter assay to analyze the promoter activity of MMP-9 and u-PA respectively. Luciferase activity, determined in triplicates, was normalized to β-galactosidase activity. The values represented the means ± SD of at least three independent experiments. *P<0.05 as compared with the vehicle group.

Testing of mRNA, reverse transcription-PCR, quantitative real-time PCR, and promoter reporter assays evaluated the inhibitory effects of NCTD on MMP-9 and u-PA expression in Huh7 cells. Huh7 cells were treated with 0, 2.5, 5, and 10 µM of NCTD for 24 h and were then subjected to RT-PCR and real time-PCR to analyze mRNA levels. MMP-9 and u-PA mRNA levels decreased considerably in a concentration-dependent manner after treatment with various concentrations of NCTD (Figs. 3A and 3B). Figures 3C and 3D show that the luciferase activities of MMP-9 and u-PA were significantly suppressed, respectively, which was determined using a luciferase assay kit. These results show that NCTD regulates the expression of MMP-9 and u-PA, at least partially, at a transcriptional level.

### NF-kB is the Key Regulator for the Transcriptional Inhibition of MMP-9 and u-PA by NCTD

**Figure 4:**
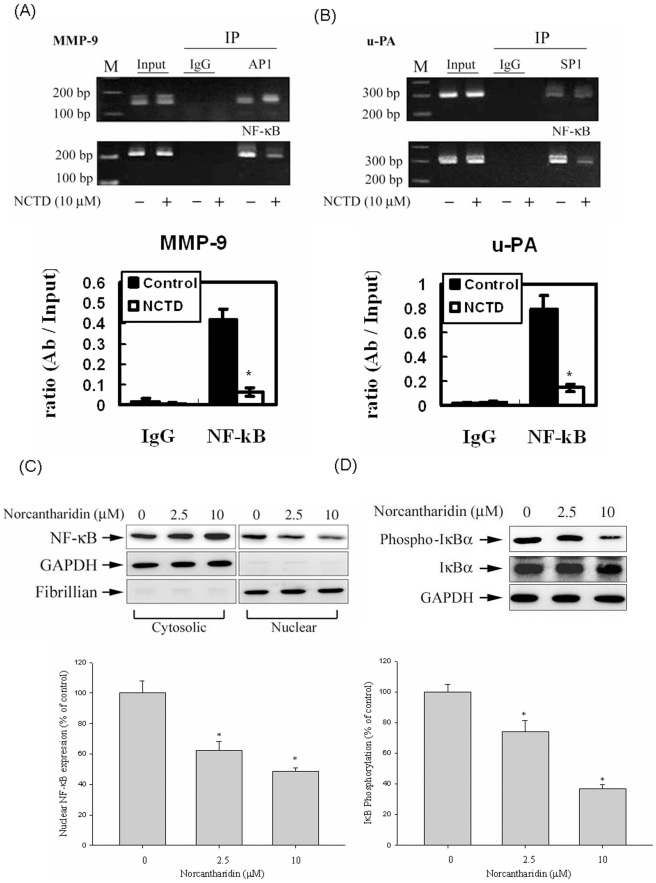
Critical role of NF-kB in NCTD-induced transcriptional inhibition of MMP-9 and u-PA in Huh7 cells. Huh7 cells were treated with NCTD 10 µM for 24 h and then the nuclear fraction was prepared as described in “*Materials and Methods*”. (A and B) ChIP analysis of the association of various transcription factors with the MMP-9 and u-PA promoter region in Huh7 cells. (C and D) Representative results of NF-KB protein levels and phosphorylation of IkBα by Western blot analysis. Quantitative results of NF-kB protein levels, which were adjusted with the internal control Fibrillian or GAPDH protein level. The values represented the means ± SD of at least three independent experiments. *P<0.05 as compared with the vehicle group.

Sequence analysis of the MMP-9 and u-PA promoter revealed a number of potential cis-acting regulatory elements, including AP-1, NF-κB, and SP-1, which could be involved in the regulation of MMP-9 and u-PA expression. We performed a chromatin immunoprecipitation (ChIP) assay to investigate the involvement of transcription factors in the transcriptional inhibitory effects of NTCD on MMP-9 (Figs. 4A to 4B). A quantitative real-time PCR assay showed that NCTD suppressed binding of NF-κB to the MMP-9 and u-PA promoters substantially (Figs. 4A to 4B).

To further test whether NF-κB is involved in the transcriptional regulation of NCTD on MMP-9 and u-PA in Huh7 cells, we evaluated the effect of NCTD on nuclear translocation of NF-κB. Treatment of Huh7 cells with 2.5 and 10 µM NTCD reduced nuclear translocation of NF-κB and phosphorylation of IkBα (Figs. 4C and 4D). The findings indicate that NCTD might induce transcriptional inhibition of MMP-9 and u-PA in Huh7 cells by suppressing NF-κB nuclear translocation and MMP-9 and u-PA-promoter binding activity.

### Effect of MAPK and PI3K/Akt pathways by NCTD

**Figure 5:**
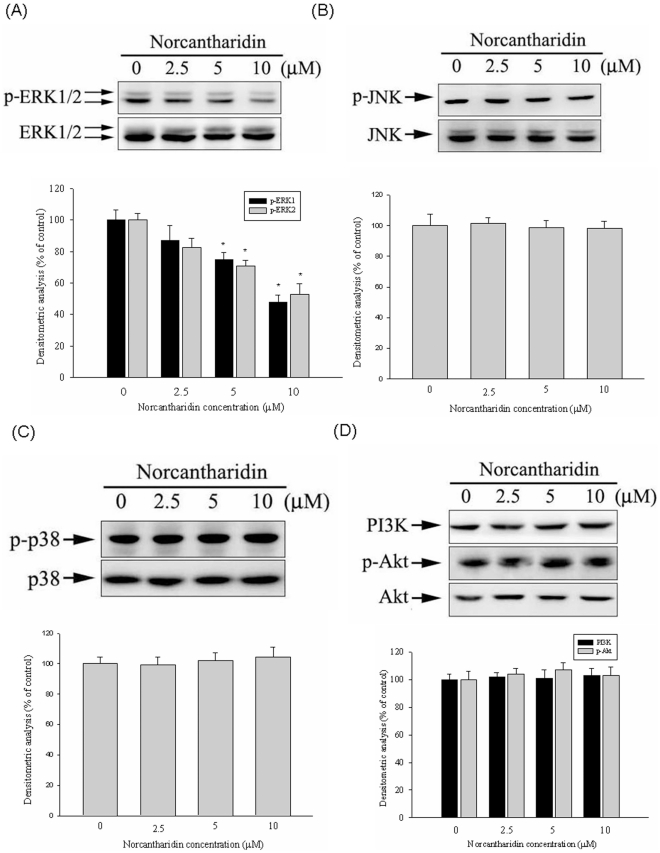
Effects of NCTD on the MAPKs pathway and PI3K/Akt signalings. Huh7 cells were cultured in various concentrations of NCTD (0, 2.5, 5, 10 µM) for 24 hours, and then the cell lysates were subjected to SDS–PAGE followed by western blots with (A) anti-ERK1/2, (B) anti-JNK, (C) anti-p38 and (D) anti-PI3K and anti-Akt (total and phosphorylated) antibodies as described in Materials and Methods. Determined activities of these proteins were subsequently quantified by densitometric analyses with that of control being 100% as shown just below the gel data. The values represented the means ± SD of at least 3 independent experiments. *P<0.05 as compared with the vehicle group.

Because the inhibitory effect of NCTD on the cell migration/invasion and proteinases was revealed, the effects of NCTD on the expressions of MAPK and PI3K/Akt pathways have been investigated by western blotting to clarify their underlying mechanisms. Western blotting showed that NCTD could reduce the phosphorylation of ERK1/2 (Fig. 5A) in Huh7 cells. According to densitometric analyses of blots versus the control, treatment of NCTD at 10 µM could result in a reduction in phosphorylation of ERK1/2 to 45%. However, phosphorylation of p38, JNK 1/2, and PI3K/Akt pathways remained unaffected (Figs. 5B–5D).

### Effect of NCTD on u-PA and MMP-9 Expression, in vitro wound closure, and Migration and Invasion of HCC cells with U0126

**Figure 6:**
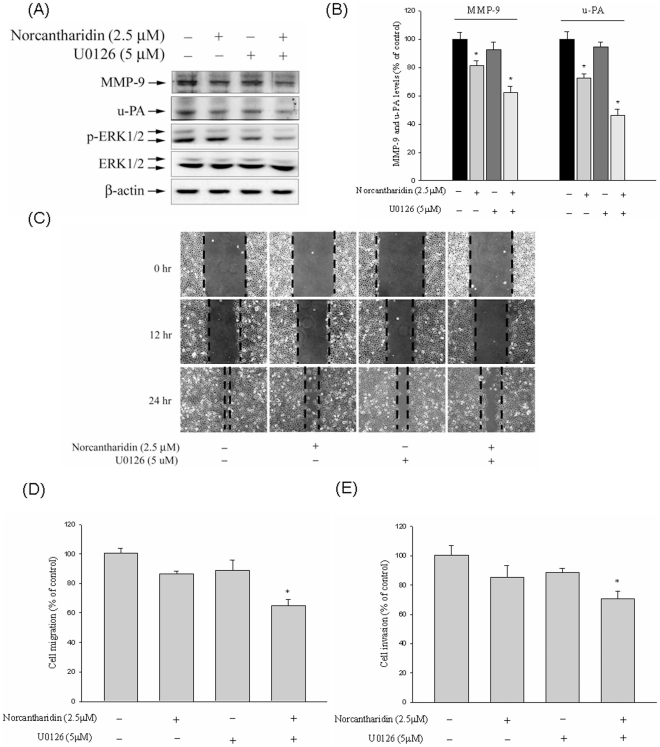
Effect of NCTD and Erk inhibitor (U0126) on MMP-9, u-PA expression, *in vitro* wound closure, cell migration and invasion in Huh7 cells. (A–B) Huh7 cells were pre-treated with U0126 for 30 min and then incubated in the presence or absence of NCTD for 24 h, and then the cell lysates were subjected to SDS–PAGE followed by western blots with anti-MMP-9, anti-u-PA, anti-Erk1/2 (total and phosphorylated) antibodies as described in Materials and Methods. (C–E) Huh7 cells were pre-treated with U0126 for 30 min and then incubated in the presence or absence of NCTD for 24, Huh7 cells were then subjected to in vitro wound closure, cell migration and invasion assay. The migration and invasion abilities of Huh7 cells were quantified by counting the number of cells that invaded to the underside of the porous polycarbonate as described in the *Materials and Methods* section. The values represented the means ± SD of at least three independent experiments. *P<0.05 as compared with the vehicle group.

To further delineate whether the inhibition of proteinase, invasion, and migration by NCTD occurred mainly through inhibition of the Erk1/2 signaling pathway, we investigated the effects of a specific inhibitor of the Erk1/2 pathway (U0126) on Huh7 cells. The results show that combined treatment of the inhibitor with NCTD further decreased MMP-9 and u-PA expression (Figs. 6A and 6B). Additionally, we observed a similar trend for the inhibition of Huh7 migration and invasion, with sole treatment and with combined treatment (Figs. 6C to 6E). Therefore, the inhibition of the Erk1/2 signaling pathways may result in reduced expression of MMP-9 and u-PA, as well as reduced tumor cell invasion.

## Discussion

NCTD has several therapeutic effects on cancer cells, including inhibition of cell proliferation, anti-angiogenic effects, cell-cycle blockage, and induction of cell apoptosis. Therefore, NCTD has been used for the treatment of primary hepatoma, esophageal carcinomas, breast cancer, gallbladder carcinomas, and leukemia [24], [25]. In hepatoma, a high concentration level of NCTD (50–100 µM) is required for a therapeutic effect on the apoptosis pathway in HCC cells, but the toxicity of NCTD remains relatively high toward normal hepatocytes. Recent studies reported that a low concentration of NCTD has anti-invasion and anti-migration effects in lung cancer A549 cells and colorectal adenocarcinoma cells [13], [18], [23], but its anti-metastatic effects in hepatoma remain unclear. Thus, we designed this study to verify whether a low concentration level of NCTD has anti-invasion and anti-migration effects in hepatoma. Our results show that a low concentration of NCTD (<10 µM) has anti-invasion and anti-migration effects in HCC cells. To our knowledge, this is the first demonstration of the anti-metastatic effect of NCTD on HCC.

In general, metastasis of cancer cells involves multiple processes and various cytophysiological changes. The degradation or breakdown of the ECM through protease is a critical step in tumor invasion or migration. This phenomenon leads to the separation of the intercellular matrix to promote cancer cell mobility, eventually leading to metastasis. Among the involved proteases, MMP-2, MMP-9, u-PA, and cathepsins reportedly play the most important roles in cancer invasion and metastasis [1]–[3]. In this study, we verified that NCTD has an inhibitory effect on metastasis through MMP-9 and u-PA in HCC cells, which is a similar conclusion of our previous research. Chen et al. demonstrated that NCTD has an inhibitory effect on metastasis because it downregulates MMP-9 expression by inhibiting Sp1 transcriptional activity in colorectal cancer CT26 cells [22]. However, no studies on anti-invasion and anti-metastasis in hepatoma exist, and the bioactive ingredients and mechanisms of NCTD remain unclear. Additionally, medicinal plants such as *Chrysanthemum indicum* and *Ganoderma lucidum* have been reported to cause downregulation of MMP-2 and MMP-9 expression in HCC metastasis with similar results to NCTD in HCC metastasis. Wang et al. showed that *Chrysanthemum indicum* ethanolic extract (CIE) suppressed the proliferation and invasion of HCC cells lines (MHCC97H cells) substantially, with notable decreases in MMP-2 and MMP-9 expression and simultaneous increases in TIMP-1 and TIMP-2 expression [26]. Weng et al. revealed that *Ganoderma lucidum* extract (GLE) contains lucidenic acid that has an anti-invasion, antiproliferative, and anti-metastatic effect on human hepatoma HepG2 cells though inhibitory effects on MMP-9 expression [27]. However, the present study's zymography data indicated that the secreted protein level of MMP-2 from Huh7 cells was quite low whereas MMP-9 expression was high. Therefore, we conclude that MMP-9 and u-PA are the most important proteases in metastasis of human hepatoma. However, a number of authors also reported that NCTD could decrease invasion through the expression of α- and β-catenin in colorectal adenocarcinoma cells and could regulate their adhesion molecules in the anti-metastatic process [23]. Thus, NCTD may use other pathways or proteases to achieve anti-metastasis in HCC, and we require more studies to substantiate any other anti-metastasis mechanisms and protease expression of NCTD.

Mitogen-activated protein kinases (MAPKs) are a family of serine/threonine kinases, including Jun-N-terminal kinase (JNK), P38, and extracellular signal-regulated kinase (ERK), which respond to chemical and physical stress by connecting cell-surface receptor responses to the activity of regulatory proteins. MAPKs activation is followed by phosphorylation of a variety of cytosolic substrates and is involved in numerous cellular programs such as cell proliferation, cell differentiation, cell invasion, cell migration, and cell death [28], [29]. Moreover, MAPK pathways in the regulation of MMPs and u-PA expression in tumor-cell invasion have been studied extensively [28]–[31]. We found that NCTD inhibits phosphorylation of ERK1/2, leading to downregulation of MMP-9 expression in HCC cells. However, other studies found that NCTD could block proliferation and induce anoikis-mediated apoptosis through the modulation of JNK/mitogen-activated protein kinase (MAPK) activation [15]. Furthermore, Luan et al. determined that NCTD treatment in A549 cell migration may inhibit the phosphorylation of Akt in lung-cancer cells [13]. Thus, the antitumor effects of NCTD may involve different pathways according to different cancer-cell types. The PI3K-Akt signaling pathway plays another crucial role in MMPs for u-PA gene regulation, cell survival, and tumor-cell invasion [2], [32]. However, Saxena NK et al. reported that concomitant activation of JAK/STAT, PI3K/AKT, and ERK signaling is involved in leptin-mediated promotion of invasion and migration of hepatocellular carcinoma cells [33]. Therefore, we require further research to confirm different pathways under varying environments.

MMPs and u-PA gene expression are primarily regulated at the transcriptional and posttranscriptional levels and at the protein level through their activators and inhibitors and cell surface localization [2], [34]. Transcription of MMPs and the u-PA gene is regulated by upstream sequences, including motifs corresponding to NF-κB, AP-1, or SP-1 binding sites [2], [34], [35]. The activation of NF-κB and AP-1 downstream of MAPK or PI3K-Akt pathways is involved in numerous pathological processes, such as inflammation, cancer-cell adhesion, invasion, metastasis, and angiogenesis [36], [37]. In this study, we found that NCTD inhibited the binding activity of NF-κB to MMP-9 and u-PA promoters in Huh7 cells, while AP-1 and SP-1 did not reduce the phenomenon. NF-κB is a transcription nuclear factor that could promote tumorigenesis, and is linked to cell invasion and metastasis. Suppression of NF-κB activation is effective in the prevention and treatment of cancer [37]. Nagendraprabhu et al. showed that astaxanthin inhibits tumor invasion by decreasing extracellular matrix production and induces apoptosis in experimental rat-colon carcinogenesis by modulating the expressions of ERK-2, NF-κB, and COX-2 [38]. Ma et al. reported that genistein potentiates have an effect similar to arsenic trioxide against human hepatocellular carcinoma by role of Akt and NF-κB [39]. Cimmino et al. showed that NCTD impairs medulloblastoma growth by inhibition of Wnt/β-catenin signaling [40]. Additionally, the phosphorylation of IκBα releases NF-κB subunits, activating NF-κB, and the subunits are then translocated from cytosol into the nucleus to regulate gene expression at a transcriptional level. Our study is the first to validate its pathway. We found that NCTD treatment results in an inhibition of NF-κB DNA-binding activity, which is accompanied by inhibition of the phosphorylation of IκBα.

In conclusion, this study showed that NCTD exerts an inhibitory effect on several essential steps of metastasis, including cell invasion and migration, by regulating the activities of metastasis-associated proteases and their natural inhibitors. The results show that NCTD may be a powerful candidate for developing preventive agents against cancer metastasis. Moreover, we demonstrated that NTCD can inhibit ERK1/2 phosphorylation effectively, by reducing NF-κB DNA-binding activities, leading to MMP-9 downregulation and u-PA expression. Clarifying signal transduction mediators and transcriptional factors involved in the NTCD anti-metastatic process on the human hepatoma cell line might effectuate the development of specific mediators to inhibit undesired cell metastasis. NTCD should be tested further *in vivo* to justify its effectiveness in the prevention of hepatoma invasion or migration.

## Materials and Methods

### Cell culture and NCTD treatment

HCC (Huh7) cells obtained from Food Industry Research and Development Institute (Hsinchu, Taiwan) was cultured in Dulbecco's modified Eagle's medium (Life Technologies, Grand Island, NY, USA), 10% fetal bovine serum, 2 mM glutamine, 100 U/mL penicillin, 100 µg/mL streptomycin, and 400 ng/mL hydrocortisone. All cell cultures were maintained at 37°C n a humidified atmosphere of 5% CO2. For NCTD treatment, appropriate amounts of stock solution of NCTD (Sigma chemical Co., St. Louis, MO, USA) were added into culture medium to achieve the indicated concentrations and then incubated with cells for indicated time periods, whereas dimethyl sulfoxide solution without NCTD was used as blank reagent.

### Determination of cell viability (MTT assay)

For cell viability experiment, a microculture tetrazolium (3-(4,5-dimethylthiazol- 2-yl)-2,5-diphenyltetrazolium bromide) colorimetric assay was performed to determine the cytotoxicity of NCTD [41], [42]. Huh-7 cells were seeded in 24-well plates at a density of 5×10^4^ cells/well and treated with NCTD at a concentration between 0 and 20 µM at 37°C for 24 h. After the exposure period, the media was removed, and cells were washed with phosphate buffered saline (PBS) and then incubated with 20 µL MTT (5 mg/mL) (Sigma chemical Co., St. Louis, MO, USA) for 4 h. The viable cell number per dish is directly proportional to the production of formazan, which can be measured spectrophotometrically at 563 nm following solubilization with isopropanol.

### In vitro wound closure

Huh7 cells (1×10^5^ cells/well) were plated in 6-well plates for 24 h, wounded by scratching with a pipette tip, then incubated with DMEM medium containing 0.5% FBS and treated with or without NCTD (0, 2.5, 5, 10 µM) for 0, 12, and 24 h. Cells were photographed using a phase-contrast microscope (×100).

### Cell invasion and migration assays

Cell invasion and migration were assayed according to the methods described by Yang et al. [43]. After a treatment with NCTD (0, 2.5, 5 and 10 µM) for 24 h, surviving cells were harvested and seeded to Boyden chamber (Neuro Probe, Cabin John, MD, USA) at 10^4^ cells/well in serum free medium and then incubated for 24 hours at 37°C. For invasion assay, 10 µL Matrigel (25 mg/50 mL; BD Biosciences, MA, USA) was applied to 8 µm pore size polycarbonate membrane filters and the bottom chamber contained standard medium. Filters were then air-dried for 5 h in a laminar flow hood. The invaded cells were fixed with 100% methanol and stained with 5% Giemsa. Cell numbers were counted under a light microscope. The migration assay was carried out as described in the invasion assay with no coating of Matrigel [44].

### Determination of MMP-9 and u-PA by zymography

The activities of MMP-9 in conditional medium were measured by gelatin zymography protease assays as previously described [43]. Briefly, collected media of an appropriate volume (adjusted by vital cell number) were prepared with SDS sample buffer without boiling or reduction and subjected to 0.1% gelatin-8% SDS-PAGE electrophoresis. After electrophoresis, gels were washed with 2.5% Triton X-100 and then incubated in reaction buffer (40 mM Tris–HCl, pH 8.0; 10 mM CaCl2 and 0.01% NaN3) for 12 h at 37°C. Then gel was stained with Coomassie brilliant blue R-250. Visualization of u-PA activity was performed as previously described [45]. Briefly, 2% casein (w/v) and 20 µg/mL plasminogen were added to 8% SDS-PAGE gel, and then performed as described in the gelatin zymography.

### RNA preparation and TaqMan quantitative real-time PCR

Total RNA was isolated from HCC cells using Trizol (Life Technologies, Grand Island, NY) according to the manufacturer's instructions. Quantitative real-time PCR analysis was carried out using Taqman one-step PCR Master Mix (Applied Biosystems). 100 ng of total cDNA was added per 25 µl reactions with MMP-9, u-PA or GAPDH primers and TaqMan probes. The oligonucleotide sequences of TaqMan probes and primers were described in Table S1. Quantitative real-time PCR assays were carried out in triplicate on a StepOnePlus sequence detection system. The threshold was set above the non-template control background and within the linear phase of target gene amplification to calculate the cycle number at which the transcript was detected.

### Preparation of total cell lysates and nuclear fraction

For total cell lysates preparation, cells were rinsed with PBS twice and scraped with 0.2 mL of cold RIPA buffer containing protease inhibitors cocktail, and then vortexed at 4°C for 10 min. Cell lysates were subjected to a centrifugation of 10,000 rpm for 10 min at 4°C, and the insoluble pellet was discarded. Nuclear extracts were obtained using a modification of a previously described method [46]. Briefly, harvested cells were scraped and lysed with buffer A (10 mM HEPES, 10 mM KC1, 0.1 mM EDTA, 1.5 mM MgCl2, 0.2% NP40, 1 m MDTT, and 0.5 m Mphenylmethylsulfonyl fluoride), followed by vortexing to shear the cytoplasmic membranes and nuclear pellets were collected by a centrifugation at 3000 rpm for 30 s at 4°C. Nuclear proteins were extracted with high-salt buffer B (20 mM HEPES, 25% glycerol, 1.5 mM MgCl2, 0.1 mM EDTA, 420 mM NaCl, 1 mM DTT, and 0.5 mM phenylmethylsulfonyl fluoride). The protein concentration of total cell lysates and nuclear fraction were determined by Bradford assay [47].

### Western blot analysis

The cell lysates or nuclear extracts were separated in a 10% polyacrylamide gel and transferred onto a nitrocellulose membrane. The blot was subsequently incubated with 5% non-fat milk in Tris-buffered saline (20 mM Tris, 137 mM NaCl, pH 7.6) for 1 h to block non-specific binding and then overnight with polyclonal antibodies against MMP-9, TIMP-1, u-PA, PAI-1, PI3K, NF-kB, Fibrillian (internal control for nuclear) or with the specific antibodies for unphosphorylated or phosphorylated activated forms of the corresponding ERK 1/2, JNK 1/2, p38, Akt and IkBα. Blots were then incubated with a horseradish peroxidase goat anti-rabbit or anti-mouse IgG for 1 h. Afterwards, signal was detected by using enhanced chemiluminescence (ECL) commercial kit (Amersham Biosciences) and relative photographic density was quantitated by scanning the photographic negatives on a gel documentation and analysis system (AlphaImager 2000, Alpha Innotech Corporation, San Leandro, CA, USA).

### Transfection and MMP-9 or u-PA promoter-driven luciferase assays

Huh7 cells were seeded at a concentration of 5×10^4^ cells per well in 6-well cell culture plates. After 24 h of incubation, pGL3-basic (vector) and MMP-9 or u-PA promoter plasmid were co-transfected with a β-galactosidase expression vector (pCH110) into cells using Turbofect (Fermentas, Carlsbad, CA). After 12 h of transfection, cells were treated with vehicle or NCTD (0, 2.5, 5 and 10 µM) for 24 h. The cell lysates were harvested, and luciferase activity was determined using a luciferase assay kit. The value of the luciferase activity was normalized to transfection efficiency and monitored by β-galactosidase expression.

### Chromatin immunoprecipitation analysis (ChIP)

Chromatin immunoprecipitation analysis was performed as described previously [46]. DNA immunoprecipitated with anti-NF-κB, anti-AP-1 and anti-SP-1 was purified and extracted using phenol-chloroform. Immunoprecipitated DNA was analyzed by PCR or quantitative PCR by using specific primers as described in Table S1.

### Statistical analysis

Statistical significances of differences throughout this study were analyzed by One-way ANOVA test to compare differences between treatments and followed up using Dunnett's multiple comparison post-hoc test. A difference at p<0.05 was considered to be statistically significant and the experiments were repeated three times.

## Supporting Information

Figure S1 **Effect of NCTD on cell viability in normal hepatocytes.** Normal hepatocytes were treated with NCTD (0, 2.5, 5 and 10 µM) for 24 h before being subjected to a MTT assay for cell viability. The values represented the means ± SD of at least three independent experiments.
(JPG)

Figure S2 **Effects of NCTD on the cell cycle regulation in Huh7 cells.** Huh7 cells were treated with NCTD (0∼10 µM) for 24 h and then subjected to flow cytometry to analyze the cell cycle regulation.
(JPG)

Figure S3 **Effects of NCTD on the cell migration in SK-Hep1 cells.** SK-Hep1 cells were treated with NCTD (0∼10 µM) for 24 h and then subjected to a Boyden chamber for 16 h with polycarbonate filters respectively. The migration abilities of SK-Hep1 cells were quantified by counting the number of cells that invaded to the underside of the porous polycarbonate as described in the *Materials and Methods* section. The values represented the means ± SD of at least three independent experiments. *P<0.05 as compared with the vehicle group.
(JPG)

Figure S4 **Effects of NCTD on the MMP-9 activity in SK-Hep1 cells.** SK-Hep1 cells were treated with NCTD (0∼10 µM) for 24 h and then subjected to gelatin zymography to analyze the activity of MMP-9. The values represented the means ± SD of at least three independent experiments. *P<0.05 as compared with the vehicle group.
(JPG)

Figure S5 **Effects of NCTD on the FAK expression in Huh7 cells.** Huh7 cells were treated with NCTD (0∼10 µM) for 24 h and then subjected to Western blotting to analyze the expression of FAK.
(JPG)

Table S1 **Primers list for real-time PCR and ChIP assay.**
(DOC)
