# Correction: Antimetastatic Effects of Norcantharidin on Hepatocellular Carcinoma by Transcriptional Inhibition of MMP-9 through Modulation of NF-kB Activity

**DOI:** 10.1371/journal.pone.0171900

**Published:** 2017-02-06

**Authors:** Chao-Bin Yeh, Ming-Ju Hsieh, Yi-Hsien Hsieh, Ming-Hsien Chien, Hui-Ling Chiou, Shun-Fa Yang

In Fig 1, Fig 1B and 1D show incorrect images under the “5” header and in the top left panel, respectively. Please view the correct Fig 1 here.

**Fig 1:**
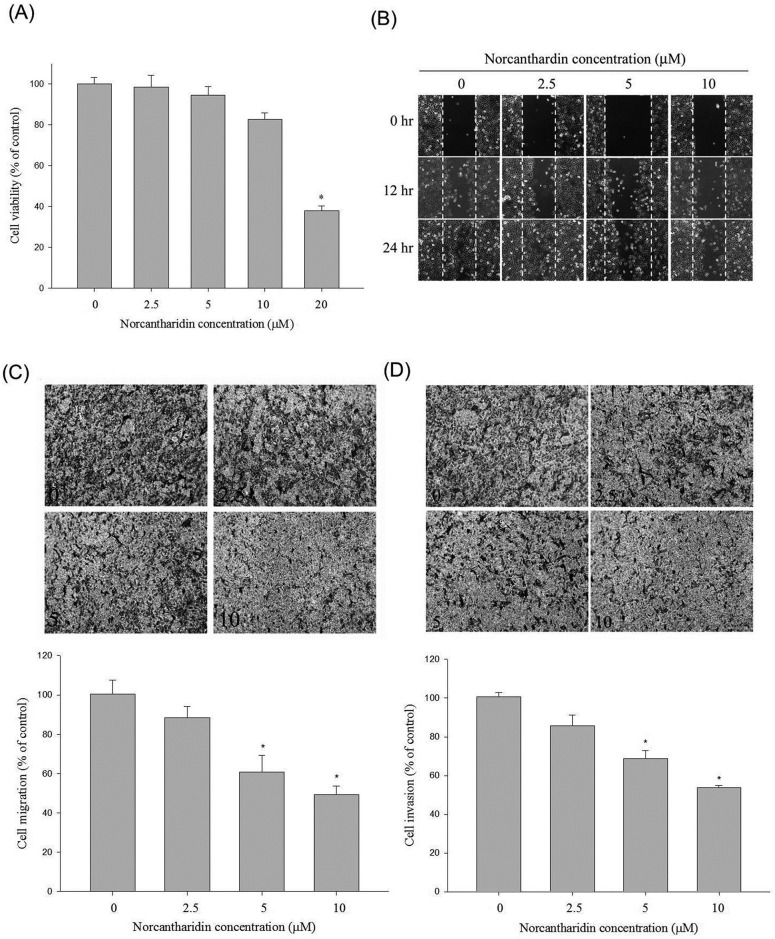
Effect of NCTD on cell viability, *in vitro* wound closure, cell migration and invasion in Huh7 cells. (A) Huh7 cells were treated with NCTD (0, 2.5, 5, 10 and 20 μM) for 24 h before being subjected to a MTT assay for cell viability. The values represented the means ± SD of at least three independent experiments. (B) Huh7 cells were wounded and then treated with vehicle (DMSO) or NCTD (2.5, 5 and 10 μM) for 0 h, 12 h and 24 h in 0.5% FBS-containing medium. At 0, 12 and 24 h, phase-contrast pictures of the wounds at three different locations were taken. (C & D) Figure C&D showing the cell migration and invasion were measured using a Boyden chamber for 16 h and 24 h with polycarbonate filters respectively. The migration and invasion abilities of Huh7 cells were quantified by counting the number of cells that invaded to the underside of the porous polycarbonate as described in the *Materials and Methods* section. The values represented the means ± SD of at least three independent experiments. *P<0.05 as compared with the vehicle group.
