# Supplementary figures and images for: Antimetastatic Effects of Norcantharidin on Hepatocellular Carcinoma by Transcriptional Inhibition of MMP-9 through Modulation of NF-kB Activity
Source: PLoS One. 2012 Feb 7;7(2):e31055. doi: 10.1371/journal.pone.0031055 (PMC3280344; doi:10.1371/journal.pone.0031055)

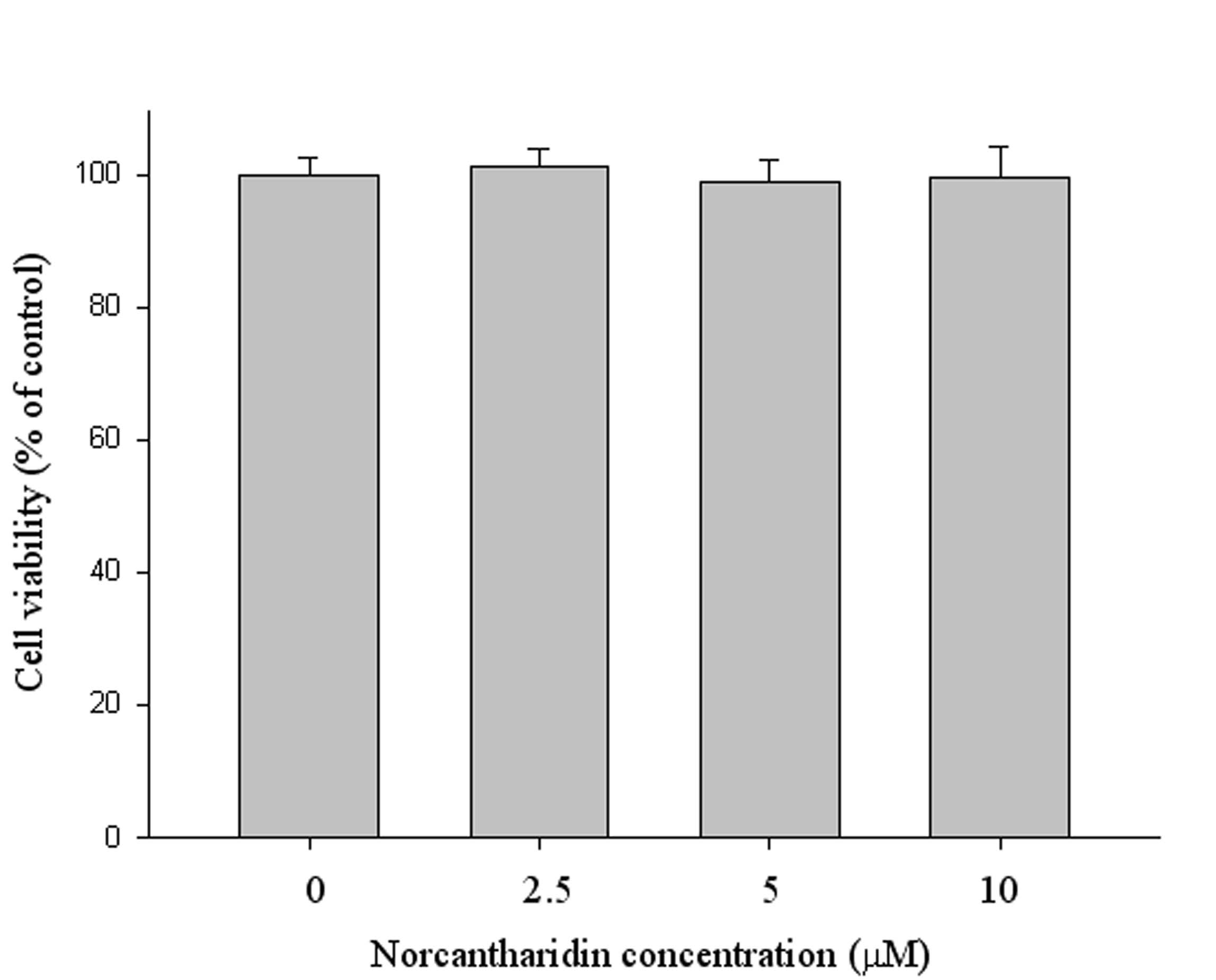

Supplement: Figure S1 — Effect of NCTD on cell viability in normal hepatocytes. Normal hepatocytes were treated with NCTD (0, 2.5, 5 and 10 µM) for 24 h before being subjected to a MTT assay for cell viability. The values represented the means ± SD of at least three independent experiments. (JPG) [file pone.0031055.s001.jpg]

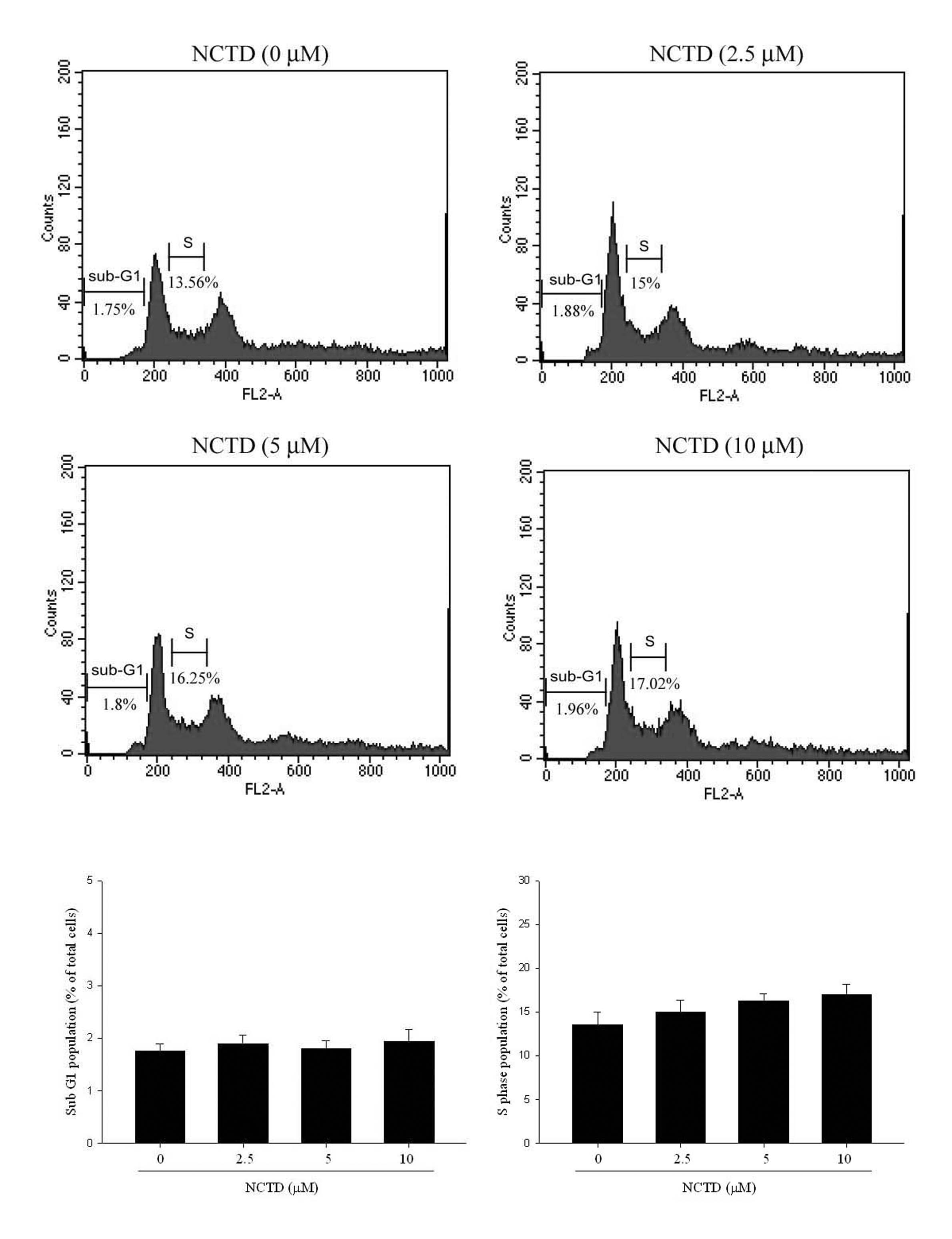

Supplement: Figure S2 — Effects of NCTD on the cell cycle regulation in Huh7 cells. Huh7 cells were treated with NCTD (0∼10 µM) for 24 h and then subjected to flow cytometry to analyze the cell cycle regulation. (JPG) [file pone.0031055.s002.jpg]

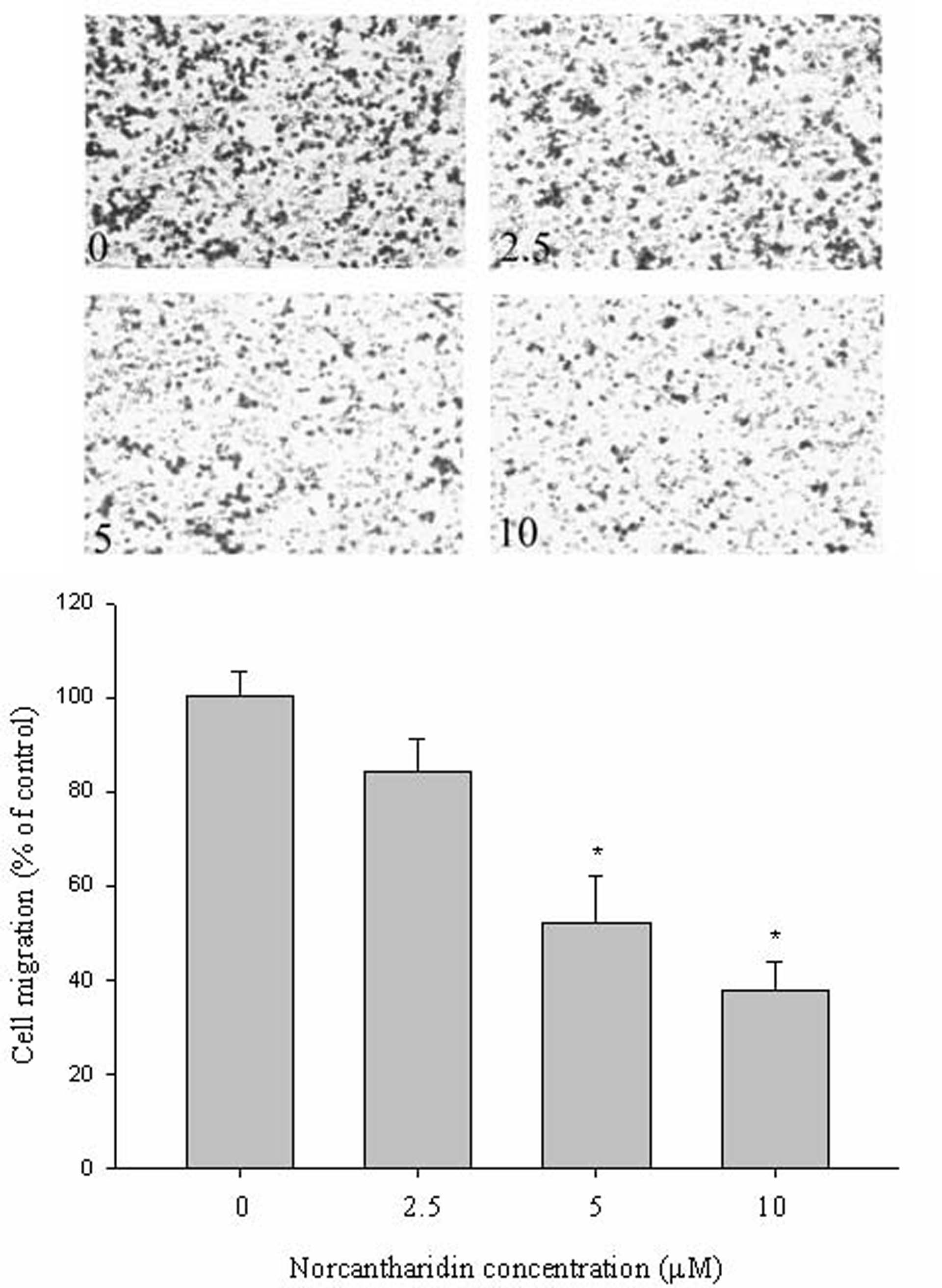

Supplement: Figure S3 — Effects of NCTD on the cell migration in SK-Hep1 cells. SK-Hep1 cells were treated with NCTD (0∼10 µM) for 24 h and then subjected to a Boyden chamber for 16 h with polycarbonate filters respectively. The migration abilities of SK-Hep1 cells were quantified by counting the number of cells that invaded to the underside of the porous polycarbonate as described in the Materials and Methods section. The values represented the means ± SD of at least three independent experiments. *P<0.05 as compared with the vehicle group. (JPG) [file pone.0031055.s003.jpg]

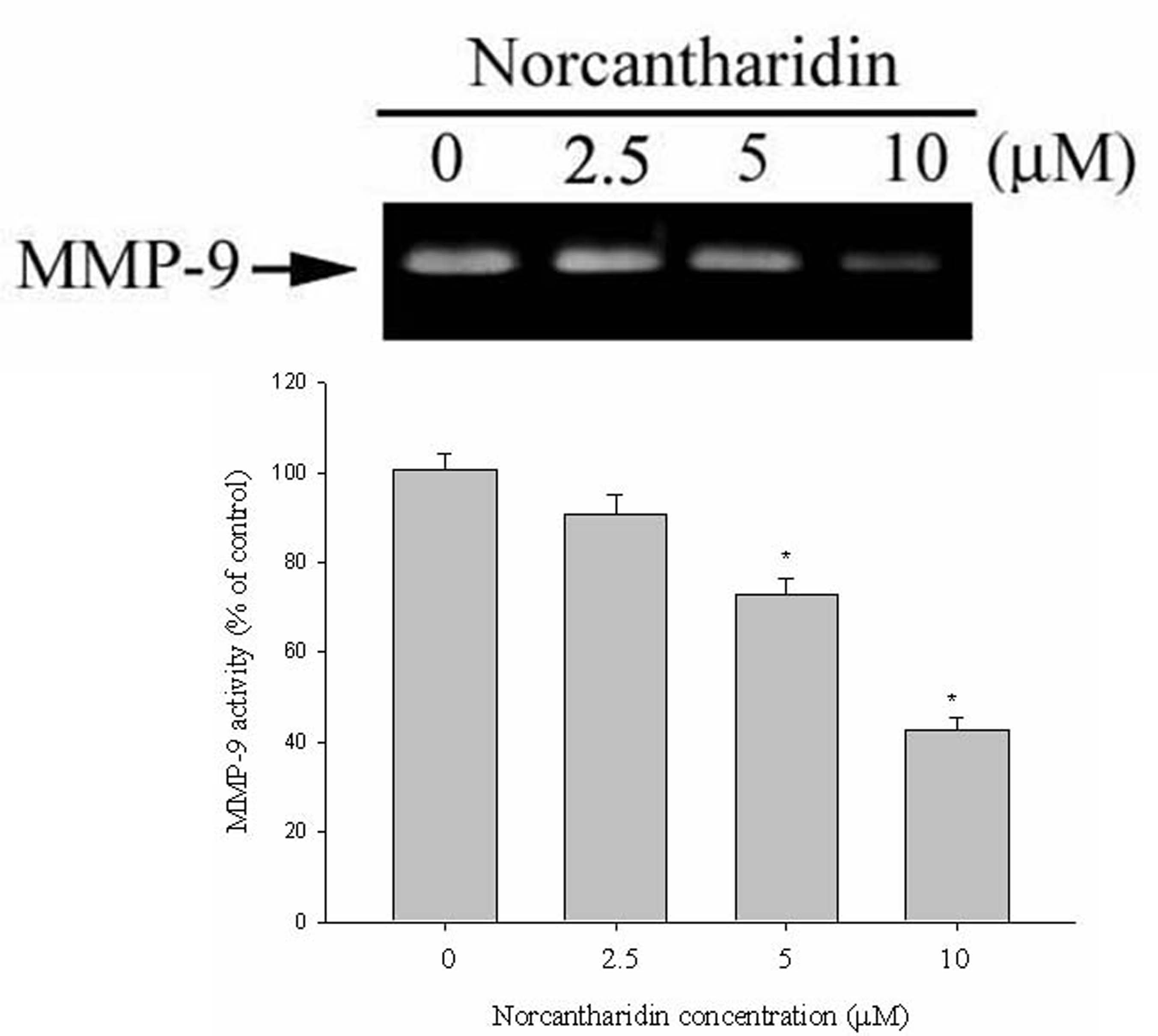

Supplement: Figure S4 — Effects of NCTD on the MMP-9 activity in SK-Hep1 cells. SK-Hep1 cells were treated with NCTD (0∼10 µM) for 24 h and then subjected to gelatin zymography to analyze the activity of MMP-9. The values represented the means ± SD of at least three independent experiments. *P<0.05 as compared with the vehicle group. (JPG) [file pone.0031055.s004.jpg]

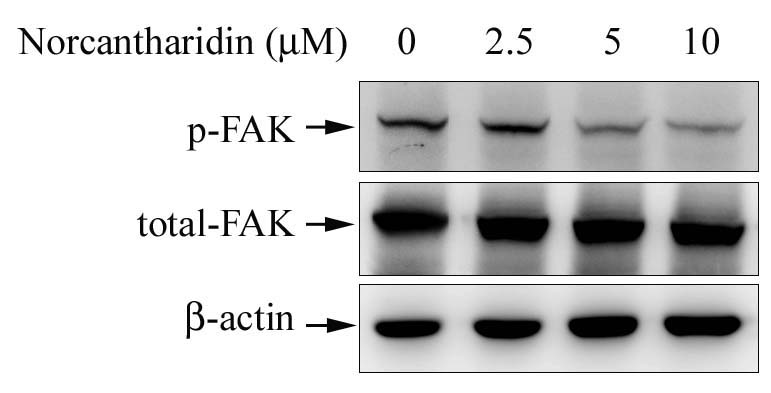

Supplement: Figure S5 — Effects of NCTD on the FAK expression in Huh7 cells. Huh7 cells were treated with NCTD (0∼10 µM) for 24 h and then subjected to Western blotting to analyze the expression of FAK. (JPG) [file pone.0031055.s005.jpg]
