# Supplementary material for: Antimetastatic Effects of Norcantharidin on Hepatocellular Carcinoma by Transcriptional Inhibition of MMP-9 through Modulation of NF-kB Activity
Source: PLoS One. 2012 Feb 7;7(2):e31055. doi: 10.1371/journal.pone.0031055 (PMC3280344; doi:10.1371/journal.pone.0031055)
Supplement: Table S1 — Primers list for real-time PCR and ChIP assay. (DOC) [file pone.0031055.s006.doc]

Table S1. Primers list for real-time PCR and ChIP assay.

| Primers used in real-time PCR | Sequence (5’ to 3’) |
| --- | --- |
| MMP-9 (Hs00957562_m1) | (FAM)- GGCGCTCATGTACCCTATGT |
| u-PA (Hs01547054_m1) | (FAM)-CAACGACATTGCCTTGCTGAAGATC |
| GAPDH ( Hs99999905_m1) | (FAM)-GGCGCCTGGTCACCAGGGCTGCTTT |
| Primers used in ChIP |  |
| MMP-9 NF-kB-F | GCCATGTCTGCTGTTTTCTAGAGG |
| MMP-9 NF-kB-R | CACACTCCAGGCTCTGTCCTCTTT |
| MMP-9 AP-1-F | TGGTGTAAGCCCTTTCTCATGCTG |
| MMP-9 AP-1-R | CAGCTGCTGTTGTGGGGGCTTTAA |
| u-PA NF-kB-F | GAGGGGGCGGAAGGGGAGAA |
| u-PA NF-kB-R | TGTGGTCAGTTTTGTTTGGATTTG |
| u-PA SP-1-F | CAGGTGCATGGGAGGAAGC |
| u-PA SP-1-R | AGGGGCGGCGCCGGGGCGG |
